# Delta-mediated cross-frequency coupling organizes oscillatory activity across the rat cortico-basal ganglia network

**DOI:** 10.3389/fncir.2013.00155

**Published:** 2013-10-02

**Authors:** Jon López-Azcárate, María Jesús Nicolás, Ivan Cordon, Manuel Alegre, Miguel Valencia, Julio Artieda

**Affiliations:** ^1^Neurophysiology Laboratory, Division of Neurosciences, CIMA, Universidad de NavarraPamplona, Spain; ^2^Department of Neurophysiology, Clínica Universidad de Navarra, Universidad de NavarraPamplona, Spain

**Keywords:** oscillatory activity, local field potentials, cross-frequency coupling, nested oscillations, nested interactions, cortico-basal ganglia network, dopaminergic system

## Abstract

The brain's ability to integrate different behavioral and cognitive processes relies on its capacity to generate neural oscillations in a cooperative and coordinated manner. Cross-frequency coupling (CFC) has recently been proposed as one of the mechanisms involved in organizing brain activity. Here we investigated the phase-to-amplitude CFC (PA-CFC) patterns of the oscillatory activity in the cortico-basal ganglia network of healthy, freely moving rats. Within-structure analysis detected consistent PA-CFC patterns in the four regions analyzed, with the phase of delta waves modulating the amplitude of activity in the gamma (low-gamma ~50 Hz; high-gamma ~80 Hz) and high frequency ranges (high frequency oscillations HFO, ~150 Hz). Between-structure analysis revealed that the phase of delta waves parses the occurrence of transient episodes of coherence in the gamma and high frequency bands across the entire network, providing temporal windows of coherence between different structures. Significantly, this specific spatio-temporal organization was affected by the action of dopaminergic drugs. Taken together, our findings suggest that delta-mediated PA-CFC plays a key role in the organization of local and distant activities in the rat cortico-basal ganglia network by fine-tuning the timing of synchronization events across different structures.

## Introduction

Neuronal oscillations exhibit large variability in amplitude and recurrence (Buzsaki and Draguhn, 2004), and they may be responsible for organizing the communication between components of large scale brain networks (Varela et al., 2001). While it is widely accepted that the coordination of different oscillatory activities plays an important role in brain processes, many aspects of the fundamental underlying mechanisms remain unknown (Singer, 1993; Canolty and Knight, 2010).

Cross-frequency coupling (CFC) has been proposed recently to be involved in the regulation of brain communication over different spatiotemporal scales. CFC provides a plausible physiological mechanism that might link activity that occurs at different rates. Phase-to-amplitude CFC (PA-CFC) has been described in humans (Canolty et al., 2006; Cohen et al., 2009; Axmacher et al., 2010; Lopez-Azcarate et al., 2010), rats (Tort et al., 2008, 2009), mice (Scheffzük et al., 2011), and monkeys (Lakatos et al., 2005; Canolty et al., 2010). Specific PA-CFC patterns have been found in different brain structures including the hippocampus (Tort et al., 2008), the basal ganglia (Tort et al., 2008, 2009; Cohen et al., 2009; Lopez-Azcarate et al., 2010), and the neocortex (Lakatos et al., 2005; Canolty et al., 2006). Both physiological and pathophysiological roles have been attributed to CFC. CFC patterns have been detected during visual motion discrimination (Händel and Haarmeier, 2009), attention selection (Schroeder and Lakatos, 2009), decision making tasks (Tort et al., 2008), and memory processes (Tort et al., 2009; Axmacher et al., 2010). Furthermore, exaggerated CFC values in the subthalamic nucleus (STN) have also been associated to the rigidity and bradykinesia seen in Parkinson's disease (PD) patients in the absence of dopaminergic treatment (Lopez-Azcarate et al., 2010).

CFC can span across space, with low-frequency phase in one area affecting high-frequency amplitude in another and *vice versa* (i.e., with the high frequency amplitude of the former being affected by the phase of the latter), thereby providing a form of frequency domain modularity that allows simultaneous communication in independent channels (Jensen and Colgin, 2007; Colgin et al., 2009). Same-structure, CFC may regulate communication between different spatio-temporal scales, while cross-structure, same-frequency coupling between different brain areas has been associated with inter-area communication (Fries, 2009; Canolty and Knight, 2010). Nevertheless, the relationship between PA-CFC and the existing measures of interaction between sites still remains unclear.

To better understand how slow activities coordinate fast oscillations over time and space, we recorded the local field potentials (LFP) at different points of the cortico-basal ganglia network of freely moving healthy rats. We characterized the CFC patterns at the local level (i.e., within each structure) and between the different structures recorded. Our results suggest that in the cortico-basal network, low-frequency delta-entrainment combined with phase-amplitude CFC provides a precise mechanism to synchronize faster activities across different frequency bands and spatial structures. Moreover, our results suggest that the dopaminergic system plays a key role in supporting such organization, which is strongly affected by the action of dopamine agonists/antagonists.

## Methods

We analyzed the oscillatory activity in the motor cortex and in three structures of the basal ganglia (caudate-putamen, CPU, STN, and subtantia nigra pars reticulata, SNr) of 15 adult male Wistar rats (250–300 g). Free-moving animals were recorded in three conditions: following saline injection (1 ml/kg) as a control condition, after the administration of 5 mg/kg of apomorphine (dopamine receptors agonist), and finally under the effect of 1 mg/kg of haloperidol (dopamine antagonist). These protocols were approved by the institutional animal ethics committee (Comité de Ética para la Experimentación Animal, Universidad de Navarra, approval ID 088-06).

### Electrode implantation

To surgically implant the electrodes, the rats were anesthetized with ketamine (75 mg/kg i.p.) and xylazine (11 mg/kg i.p.), and then situated in a stereotaxic frame using blunt ear bars to avoid any damage to the animals' tympanic membrane. The target coordinates for electrode placement were selected according to the Paxinos and Watson atlas: anterior (AP) 2.20 mm and lateral (L) 3.20 mm for the motor cortex; AP: −4.8 mm and L: 7.4 mm for the auditory cortex (reference for motor cortex recording); AP: 0.20 mm and L: 3 mm, ventral, V: −6 mm for the caudate-putamen; AP: −3.80 mm, L: 2.5 mm, V: −7.8 mm for the STN; and finally, AP: −5.80 mm, L: 2 mm, V: −8 mm for the SNr.

Two different types of electrodes were used to record LFP from the aforementioned brain structures. Concentric microelectrodes with two contacts (inner contact area 0.157 mm^2^, outer contact area 0.393 mm^2^, Model SNE-100, Kopf Instruments, Tujunga, California, USA) were placed stereotactically in the CPU, STN, and SNr, while cortical LFP were recorded by means of stainless steel screws placed in the skull (1.6 mm diameter, Plastics One, Roanoke, VA, USA, Ref. E363). The active electrode was placed in the primary motor cortex and was referenced to an electrode placed in the auditory cortex. An additional screw placed in the frontal region was used as the ground electrode. The wires of the electrodes were connected to a custom-made small ten-channel socket that was firmly fixed to the rat's skull with dental cement (Faciden, Olot, Spain). The skin only left the terminal male pins of the socket uncovered.

Antibiotic was administrated orally over 1 week to avoid infections (enrofloxacin, Alsir 10%: Esteve, Spain) and postoperative intramuscular analgesics were also administrated (Ketoprophen, 2 mg/kg sc, Ketofen 1%: Lab, Spain). Pharmacological experiments began 5 days after surgery.

### Recording

The animals were recorded inside a custom-made Faraday cage shielded from external electrical fields and they were connected to the recording equipment by two cables that hung from the top of the cage (Ref. 363-363 50 cm 6TCS, spring: Plastics One, Roanoke, VA, USA). A multi-channel rotary commuter was used to allow the animals to move freely inside the cage (SL12C/SB: Plastics One, Roanoke, VA, USA). The recording procedure commenced 5–7 days after electrode implantation and it began 45 min after connecting the cables in order to let the animals habituate to the Faraday cage.

All recordings were carried out in the same order in each animal over 3 days. On the first day the animals were recorded after saline injection (i.p., 1 ml/kg; 0.9%, B Braun, Barcelona, Spain), while on the second day the oscillatory activity was studied under the effect of apomorphine (i.p., 5 mg/kg, Apo-go Pen, Italfarmaco, Madrid, Spain) and on the third day the animals received a bolus of haloperidol (i.p., 1 mg/kg Haloperidol, Esteve, Barcelona, Spain). Two animals did not receive haloperidol injection, and one animal was only recorded after saline administration and was not analyzed further.

Animals' movement was tracked using a webcam located at the top of the cage during the whole recording time and semi-automatically quantified by means of custom-made routines running under Matlab (Mathworks, Natick, MA, USA).

### Signal conditioning

The signals were filtered at 0.3–1000 Hz, amplified 20000-fold using Grass P511 amplifiers (Grass, W Worwick, RI, USA), sampled at 2500 Hz, and stored in a personal computer using Spike2 software and a CED 1401 power analog-to-digital converter (Cambridge Electronic Design).

Misplaced electrodes (detected in the histological analysis) and recording channels with suboptimal signals were not analyzed further, giving a total of: 14 motor cortex, 14 CPU, 10 STN, and 10 SNr recordings studied in the saline group; 14 motor cortex, 14 CPU, 10 STN, and 11 SNr recordings analyzed under the effect of apomorphine; and 12 motor cortex, 12 CPU, 8 STN, and 8 SNr recordings studied in the haloperidol injected group.

For each animal and condition, 300 s segments free of artifacts were selected for further analysis.

All the data analysis procedures were performed by the use of custom-made software running under Matlab (Mathworks, Natick, MA, USA).

### Power spectrum analysis

The frequency content of the signals were characterized by means of the Welch periodogram (Halliday et al., 1995) using a fast Fourier transform 4 s long and a Hanning window, giving a resolution of 0.25 Hz per bin. Power spectra for each channel, treatment, and animal were computed and evaluated as described previously (Valencia et al., 2012).

### Cross-frequency interactions

Cross-frequency interactions between different frequency bands were assessed by means of the modulation index [MI: as outlined in (Tort et al., 2008)]. First, raw data was filtered in two frequency ranges of interest (low-frequency and high-frequency) before the instantaneous phase from the low-frequency filtered data (*ϕ*) and the instantaneous amplitude (envelope) of the high frequency filtered data (*A*) were computed by means of the Hilbert transform. Finally, the coupling between the phase of the slow wave and the amplitude of the fast activity was determined by the entropy of the distribution obtained by computing the mean amplitude of the instantaneous envelope (*A*) within *n* phase intervals of the instantaneous phase (*ϕ*).

A normalized entropy index was computed *MI* = (H_max_ −H)/H_max_ where *H*_max_ = log (*n*) is the maximum entropy $H=\;-\sum_{i\;=\;1}^{n}p_{i}\log(p_{i})$ and $p_{i}=\frac{A(\phi_{i})}{\sum_{i\;=\;1}^{n}A(\phi_{i})}$ is the normalized amplitude of the fast oscillation at each of the *n* phase intervals considered (here we set *n* = 18 intervals). When the MI is 0, there is no phase-to-amplitude modulation and the phase-amplitude distribution is uniform, whereas larger MI values arise from stronger modulation. The statistical significance of the MI values was assessed by computing 200 surrogates, adding a temporal random offset to the amplitude signal. Assuming that the surrogate data follow a normal distribution, a Z-score value can be computed and used to estimate the significance of the MI parameter.

Specifically, here we computed the phase signal by filtering the raw signal from 0.5 to 12 Hz in 0.25 Hz steps with a 1 Hz bandwidth, and the amplitude signal by filtering from 12 to 200 Hz in 2 Hz steps with a 4 Hz bandwidth.

We also estimated the time–frequency plots of the mean normalized power of the high-frequency activity (12–200 Hz) locked to the phase troughs of the delta-theta activity (2–8 Hz), as indicated elsewhere [(Canolty et al., 2006) see Figure 4, second row]. Segments of the raw signal were also averaged, centering on the peaks of the three high-frequency bands (low-gamma, high-gamma, and HFO). A minimum 500 ms separation between peaks was used to avoid selecting more than one peak in a delta cycle (125 ms for the theta range, see Figure 4, third row).

### Imaginary coherence

Functional relationships between the motor cortex and the basal ganglia nuclei were estimated by means of the imaginary coherence (iCoh). This measure derives from the coherency and has been shown to be insensitive to false connectivity arising from volume conduction, identifying only true brain interactions over different frequency bands and reflecting the oscillatory properties of the brain electrical activity (Nolte et al., 2004).

Coherency is a complex measure whose magnitude (coherence) and phase can be used to assess the existence of functional interactions. It can be shown that the coherency of non-interacting sources is necessarily real, and hence, the imaginary part of coherency provides an excellent candidate to study true interactions. It is only sensitive to the synchronization of two processes that are time lagged to each other. Thus, as volume conduction does not cause a time lag, the imaginary part of coherency is insensitive to this artifactual self-interaction.

Here, we used a sliding window of 4 s with no overlap in order to compute the iCoh. The statistics were derived from the transformed iCoh values (z-transformation) as described in Halliday et al. (1995); Nolte et al. (2004).

### Phase-locked (evoked) and non phase-locked (induced) changes in oscillatory activity

In order to study the presence of induced or evoked changes in the oscillatory activity recorded in these structures, we first detected the time points corresponding to the phase troughs of the delta (theta) activity and selected 2-s segments. Three different mathematical approaches were then applied to the time-frequency (TF) decomposition of the signals recorded (Valencia et al., 2006). We used the Stockwell transform (ST), a complex transformation that gives an accurate estimate of the varying energy and phase of the signal analyzed in a selected time–frequency window of interest (Stockwell et al., 1996). The ST is suitable for this study because it provides a unique time–frequency representation of a signal by adapting the Fourier transform to analyze a localized signal using frequency-dependent time-scaling windows, thereby allowing us to study a wide range of frequencies (specifically 20–200 Hz, setting the width factor of the localizing Gaussian to a value of 7). In fact, the ST is a short-time Fourier transform with scalable Gaussian windows that provides a frequency-dependent resolution.

In the first approach, we studied the time–frequency transform of the averaged signal in a 2-s window centered on the troughs of the delta (theta) waves (see Figure 6, first row). All sweeps from each animal and condition were averaged in order to obtain the classic waveform of the evoked potential (EP) and the square of the ST-spectrum amplitude was then calculated, giving us the TF representation of the EP energy for the frequencies and time-slot of interest (STAvg).

In second place, by averaging the magnitude of single-sweep ST we obtained an estimate of the variation of the spectral content in the time period analyzed (2 s windows centered on the troughs of the delta waves) considering only the changes in amplitude and excluding the influence of the phase (see Figure 6, second row). To do that, complex coefficients were obtained for every single sweep, ST(t,f), their absolute values were squared to obtain power estimates, and finally they were averaged across the different sweeps (AvgST).

Ultimately, the inter-trial coherence [ITC: (Makeig et al., 2002)] was assessed through the phase of the ST-spectrum. The ITC(t,f) values range from 0 to 1:0 for purely non-phase locked activity and 1 for strictly phase-locked activity (see Figure 6, third row). With this measure, even very low-amplitude signals can be detected provided that they are phase-locked across trials. In this approach we also used 2 s windows centered on each trigger.

To assess the statistical significance of these analyses, we computed a distribution of 200 surrogate data for every single recording, channel and approach, adding a temporal random offset to the trigger time points. Assuming that the surrogate data follow a normal distribution, we then transformed the STAvg, AvgST, and ITC values into a N(0,1) distribution where we could evaluate their significance.

### Event related coherence (ERCoh) and phase locking value (PLV) analysis

Event-related coherence (Andrew and Pfurtscheller, 1996) was calculated between the motor cortex and the three basal ganglia structures recorded, in 2 s windows that were centered on the troughs of the delta (theta) waves. While coherence provides a normalized measure of the linear correlation between signals in the frequency domain, the ERCoh calculates a time-dependent estimate of the coherence related to a specific event (the trigger). As both the amplitude and the phase of the signals affect this analysis we also used the phase locking value (PLV). This measure only accounts for the phase, thereby allowing us to separate amplitude related activation from strictly phase-related phenomena (Lachaux et al., 1999).

In order to assess the statistical significance of these analyses, we also computed a distribution of 200 surrogate data for every single recording, pair of channels and approach. These data were generated by selecting the segments of each channel from a different random permutation of the trigger time points (troughs of the slow waves). Assuming that the surrogate data follow a normal distribution, we can then obtain a threshold value for the significance of both the ERCoh and PLV values.

### Statistics

In order to test for statistical differences between the phase-to-amplitude coupling patterns described, we compared the frequency of the modulating activity, the frequency of the modulated activity (in the low-gamma, high-gamma, and HFO bands) and the magnitude of the MI measured in each rat, nuclei and condition (saline, apomorphine, and haloperidol).

The distribution of the different variables was studied prior to any statistical analysis and those not following a normal distribution were normalized using the transformation described previously (van Albada and Robinson, 2007). Briefly, this transformation is based on the fact that a uniform distribution can be obtained from any continuous variable by calculating its cumulative distribution function (CDF). Thus, the normalization of non-gaussian variables can be reached by applying a function (inverse error function) that transforms a uniform distribution to a normal distribution.

In the first place, a Two-Way repeated measures ANOVA (structure × frequency) was used to compare the MI values across different structures and frequencies in the control condition. Secondly, the effect of dopaminergic modulation on the frequency of the modulating activity was assessed by means of a Two-Way ANOVA (drug × structure). Similarly, the effect of dopaminergic drugs on the frequencies of the modulated oscillations was assessed by means of three independent (one per band) Two-Way ANOVAs (drug × structure). Finally, a Three-Way repeated measures ANOVA (drug × structure × frequency) was used to account for the differences in the MI strength depending on the drug, structure recorded or frequency of coupling.

In all cases, Bonferroni corrected *t*-tests were used as *post-hoc* analysis. Missing values were imputed with the mean values obtained for the control condition, as the results were identical when an analysis based on multiple imputations was performed.

## Results

We analyzed the LFP oscillatory activity in the motor cortex, caudate putamen (CPU), STN, and substantia nigra pars reticulata (SNr) of a group of freely moving, healthy rats in control conditions (saline, 1 ml/kg), under the effects of a dopamine agonist (apomorphine, 5 mg/kg), and after the injection of a dopamine antagonist (haloperidol, 1 mg/kg).

### Power spectral analysis

**Figure 1:**
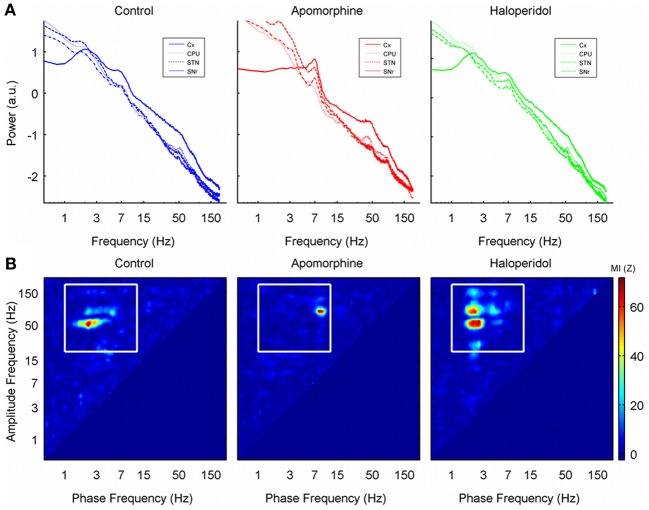
**(A)** Grand-average of the power spectrum in the 0–200 Hz range (log–log scale) for the four structures recorded in each of the three conditions studied. Note the 1/f characteristic of the power spectrum. There are clear peaks in the delta, theta, and gamma ranges of the spectra in the saline and haloperidol conditions. Low-gamma activity (~50 Hz) is attenuated by apomorphine and is replaced by a peak around 40 Hz together with an increase in power of the high-gamma activity (~80 Hz). **(B)** Cross frequency coupling co-modulograms (in terms of the MI z-score values) of one single animal (rat 8, CPU) in the 0–200 Hz frequency range after the administration of saline, apomorphine, and haloperidol. Note that the phase of delta oscillations (~3 Hz) significantly modulates the amplitude of gamma and high frequency oscillations in control and haloperidol conditions, while the modulating frequency shifts to the theta range after apomorphine administration (~6 Hz). No oscillations faster than 10 Hz significantly modulate the amplitude of other activities.

The results of power spectral analysis have already been presented elsewhere (Valencia et al., 2012). Briefly, all the structures recorded showed a power spectrum that followed a straight line when plotted in log–log coordinates (called the *power-law characteristic* or *fractal component* of the power spectrum), with a number of peaks (called *oscillatory modes*) superimposed. The control condition was characterized by the presence of peaks in the delta (~3 Hz), theta (~6 Hz) gamma (low-gamma, ~50 Hz; high-gamma, ~80 Hz) and high frequency ranges (HFO, ~150 Hz: Figure 1A, blue traces). Dopaminergic modulation generated changes in both the slope of the power law and in the peaks. Apomorphine administration decreased the slope of the power law characteristic, it increased the amplitude of the oscillations in the theta and high-gamma ranges, and it displaced the frequency of the low-gamma band activities from ~50 to ~40 Hz. (Figure 1A, red traces). Haloperidol administration increased the slope of the spectra but it did not produce any change in the amplitude of the peaks (Figure 1A, green traces).

### Phase-amplitude CFC patterns in the cortico-basal ganglia network structures

To determine whether PA-CFC interactions across these frequency peaks occurred and to evaluate the effects of dopaminergic modulation, we computed the co-modulogram of the signals in the 0–250 Hz range. In the four structures analyzed, co-modulograms detected the presence of a strong PA-CFC between the phase of activities in the delta range (theta for apomorphine condition, see below), and the amplitudes in the gamma and HFO bands. No oscillatory activities faster than those in the delta (theta) range modulated the amplitude of any other oscillations (see Figure 1B).

**Figure 2:**
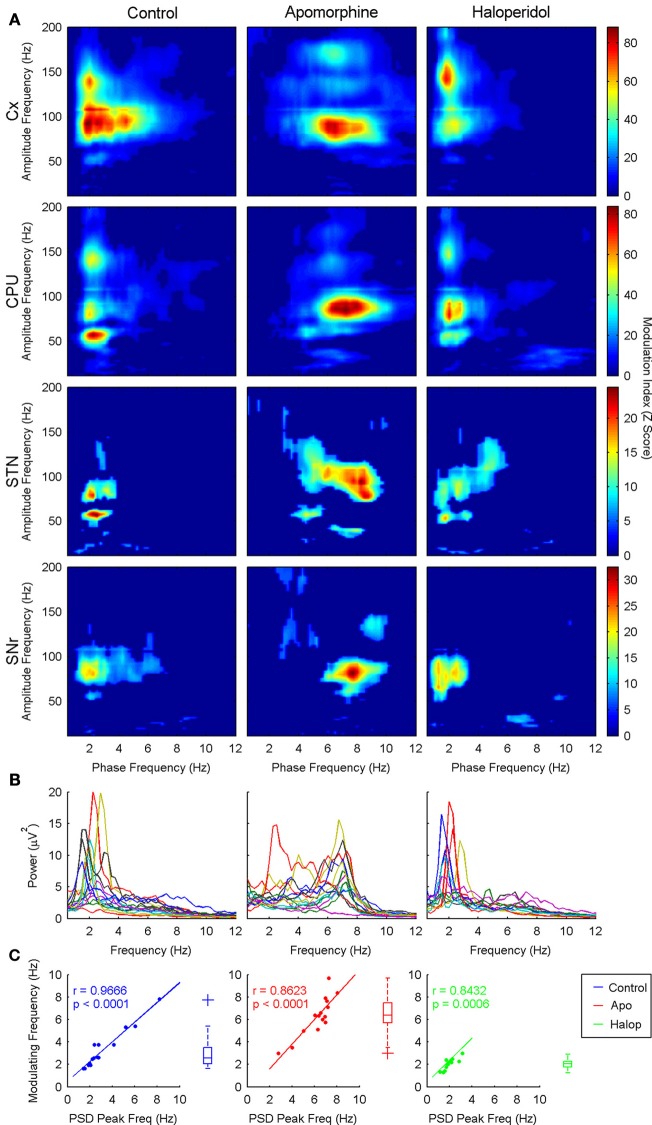
**(A)** Grand-average of the co-modulograms (shown as MI z-score) showing cross-frequency coupling interactions between the phase of the delta activity (theta for apomorphine) and the amplitudes of the low-gamma, high-gamma, and HFO bands across the four structures recorded in the three conditions studied. In saline and haloperidol conditions the phase of the delta activity entrains the amplitude of the low-gamma, high-gamma, and HFO bands, with a distinct configuration depending on the structure (see text). Apomorphine administration induces a shift in the modulating activity into the theta range. Under the effects of apomorphine this coupling pattern is similar in the four structures, revealing a predominant theta-to-high-gamma coupling. Only statistically significant phase-to-amplitude cross-frequency coupling values are shown (Z-score > 5, *p* < 0.001, Bonferroni corrected). **(B)** Power spectrum density in the 0–12 Hz range in the three conditions studied (each line represents a different animal). Note the presence of a marked delta peak in both saline and haloperidol conditions, while under the effects of apomorphine the predominant peak is in the theta range. **(C)** Delta (theta for apomorphine) peak frequencies measured in the power spectrum density are significantly correlated with the modulating frequency values across the different animals in the three conditions studied. Boxplot diagrams represent the distribution of the modulating slow frequency values.

**Figure 3:**
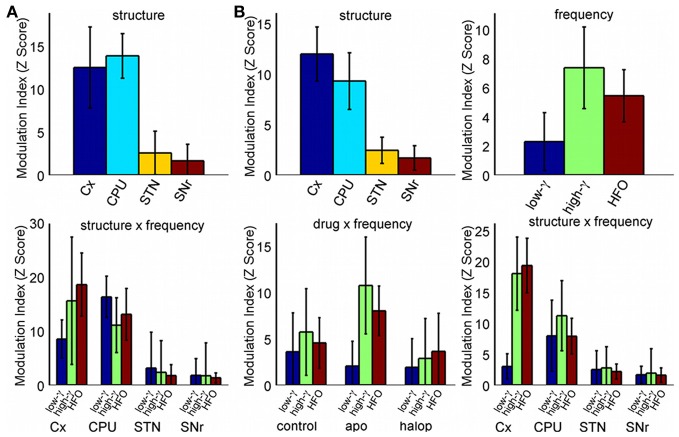
**Distribution of the Z-score MI values (median and standard error of the median). (A)** MI values for the different structures and frequencies in the control condition (Two-Way ANOVA). **(B)** MI values including the drug effect (Three-Way ANOVA). Only significant effects in ANOVAs are shown.

A more detailed estimate of the co-modulograms was computed in the (0.5–12) Hz and (12–200) Hz frequency ranges for the phase and amplitude signals, respectively (Figure 2A). In the control condition, co-modulograms were characterized by a strong PA-CFC pattern, with the phase of delta oscillations (mean frequency, 3 Hz; *SD*, 1.37 Hz) modulating the amplitude of activities in the low-gamma (mean, 54.75 Hz; *SD*, 7.46 Hz), high-gamma (mean frequency, 90.29 Hz; *SD*, 11.88 Hz) and HFO frequency bands (mean frequency, 142 Hz; *SD*, 14.34 Hz). Differences in the MI strength were assessed by a repeated measures Two-Way ANOVA (structure × frequency), which identified significant effects for the structure factor [*F*_(3, 39)_ = 6.52; *p* < 0.01] and for the interaction term [*F*_(6, 78)_ = 2.98; *p* < 0.05]. Bonferroni *post-hoc* tests indicated that MI values in the cortex and CPU were significantly larger than those in STN or SNr (*p* < 0.05, with no differences among them). In addition, MI values in the SNr were lower (*p* < 0.05) than those in the STN (see Figure 3A, top). The interaction effect reflected that MI values in the cortex were stronger in the highest frequency ranges (showing significant differences between the HFO and the low-gamma values) while in the basal ganglia nuclei MI values tended to be higher in the gamma range (see Figure 3A, bottom).

#### Effects of dopaminergic modulation

Dopaminergic modulation induced noticeable effects in the PA-CFC patterns. Apomorphine and haloperidol affected the modulating frequency, the characteristics of the modulated activities (frequency and MI strength), as well as the preferred phase of modulation.

The animals moved more after the administration of apomorphine than after the injection of saline or haloperidol [One Way ANOVA with drug factor, *F*_(2, 36)_ = 17.9; *p* < 0.001]. Nevertheless, no differences were detected when comparing saline vs. haloperidol conditions.

***Shift of the modulating frequency.*** The values of the modulating frequency were significantly different in the three conditions analyzed. two-way anova (drug × structure) revealed a significant effect of the drug factor [*f*_(2, 74)_ = 63; *p* < 0.001], while no differences related to structure or interaction effects were detected. bonferroni *post-hoc* tests revealed that value of the modulating frequency under the action of apomorphine (Figure 2a, second column) was significantly higher (*p* < 0.05) than that obtained in the control condition (Figure 2a, first column) or after haloperidol injection (Figure 2a, third column). actually, apomorphine administration induced a shift of the modulating frequency from the delta to the theta frequency range (mean frequency, 6.42 hz; *sd*, 1.51 hz). by contrast, haloperidol significantly decreased the modulating frequency (mean frequency, 2.02 hz; *sd*, 0.41 hz, *p* < 0.05) with respect to the control condition.

Moreover, the values of the modulating frequencies (measured in the phase-to-amplitude co-modulograms) were correlated with those of the spectral peaks presenting the maximal power in the delta/theta range (Figures 2B,C).

The values of the modulating frequency also correlated with the locomotor activity of the animals but only after apomorphine administration (Pearson linear correlation coefficient, *r* = 0.543; *p* < 0.001). Interestingly, no significant correlation was detected between the modulating frequency and the animals' movement after saline and haloperidol administration.

***Effect on the CFC pattern.*** After apomorphine administration, the co-modulograms showed significant coupling patterns between the phase of the slow waves (now in the theta range) and the three frequency bands described above (i.e., low-gamma, high-gamma, and hfo). apomorphine not only changed the frequency of the modulating oscillations but also, the frequency of the modulated activity in the low-gamma range: two way-anova (with drug × structure factors) detected significant effects for the drug factor on the values of the low-gamma frequency [*f*_(2, 54)_ = 8.39; *p* < 0.001]. no structure effects were detected for any of the three frequency bands. consistent with the spectral shift caused by apomorphine, bonferroni *post-hoc* tests showed that the gamma frequency values in the theta-to-low-gamma pair were significantly lower (*p* < 0.05, mean frequency, 43.18 hz; *sd*, 9.77 hz) than under haloperidol or control conditions. haloperidol did not exert any changes in the central frequency of the low-gamma activities coupled to the phase of the slow (delta) waves.

In the analysis of the strength of the MI, a Three-Way repeated measures ANOVA (drug × structure × frequency) detected a significant effect for the factors structure [*F*_(3, 39)_ = 14.9; *p* < 0.001], frequency [*F*_(2, 26)_ = 3.98; *p* < 0.05] and for the (drug × frequency) [*F*_(4, 52)_ = 5.04; *p* < 0.05] and (structure × frequency) [*F*_(6, 78)_ = 6.46; *p* < 0.001] interactions. No effects were detected for the (drug × structure) or the (drug × structure × frequency) interactions.

The *post-hoc* analysis of the structure factor showed again that the largest values of MI were present in the Cx and CPU (*p* < 0.05; with no differences between them) and the lowest in the SNr (*p* < 0.05: Figure 3B, top left). *Post-hoc* tests for the frequency factor detected that the strongest values of MI were observed in the high-gamma band, with a significant difference between the high-gamma and low-gamma ranges (*p* < 0.05), and showing a trend for the high-gamma/HFO comparison (*p* ~0.1: Figure 3B, top right). The (drug × frequency) interaction factor can be explained by the changes induced by apomorphine in both gamma bands. With respect to the control condition, apomorphine increases the MI values in the high gamma band (actually, these values are the largest all over the conditions, Bonferroni *post-hoc* test, *p* < 0.05) while it decreases those in the low-gamma range, thereby generating a significant difference between them. By contrast, haloperidol only demonstrates a trend (*p ~ 0.1*) toward a decrease in the coupling of the two gamma bands (Figure 3B, bottom left).

The (structure × frequency) interaction reflects the differences in the distribution of the MI values across the different structures in function of the frequency. While the CPU has the highest values of MI for the low-gamma band, the cortex has the strongest MI values for the high-gamma and HFO frequency bands, followed by the CPU, STN, and the SNr. In the cortex, coupling values in the low-gamma band are significantly lower than those in the high-gamma bands and HFO (Bonferroni *post-hoc* test, *p* < 0.05), while no differences were observed across frequencies in the CPU, STN, or SNr (Figure 3B, bottom right).

Pearson linear correlation coefficient detected a significant correlation between the strength of theta/high-gamma PA-CFC and the locomotion activity showed by the animals after apomorphine injection (*r* = 0.555; *p* < 0.001). No significant correlations were found for the other two bands and/or conditions.

Together, these results suggest that modulation of the dopaminergic system (i.e., the action of apomorphine and haloperidol) not only induces a shift in the modulation frequency of the PA-CFC patterns but also it exerts differential effects on the strength of modulation depending on the structures recorded and the frequencies involved.

**Figure 4:**
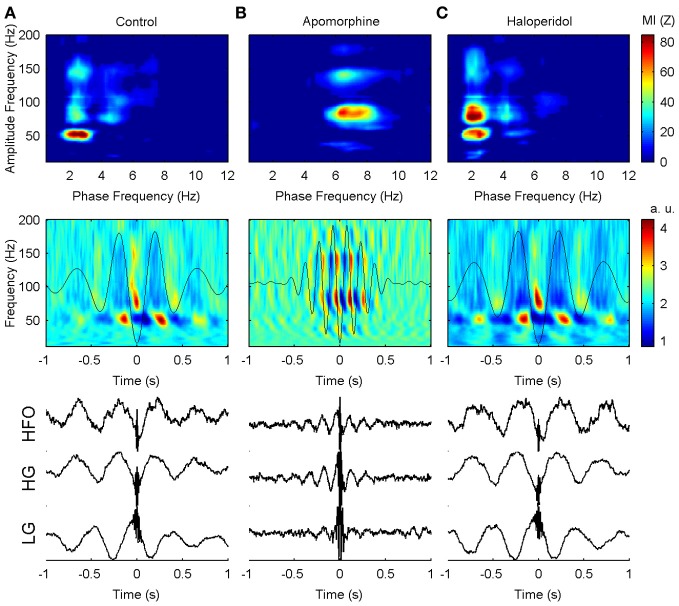
**Example of the phase-to-amplitude coupling of the oscillations recorded in the CPU of a single representative animal (rat 9) in the three conditions studied. (A)** In the control condition, low-gamma, high-gamma, and HFO are modulated by delta activity (upper panel) while in a different phase of the delta cycle (middle and lower panels). By contrast, low-gamma oscillations predominate in the peak and descending phase of the delta oscillation, while high-gamma and HFO activity are maximal in the valley of the delta cycle. **(B)** After apomorphine administration the amplitude of low-gamma, high-gamma, and HFO activities is modulated by a faster modulating activity in the theta range (upper panel). The amplitudes of all these activities peak in the same phase (peak) of the theta waves (middle and lower panels). **(C)** Delta-to-fast oscillation coupling after haloperidol injection is very similar to that in saline conditions, with only a slightly lower frequency of the modulating delta activity.

**Figure 5:**
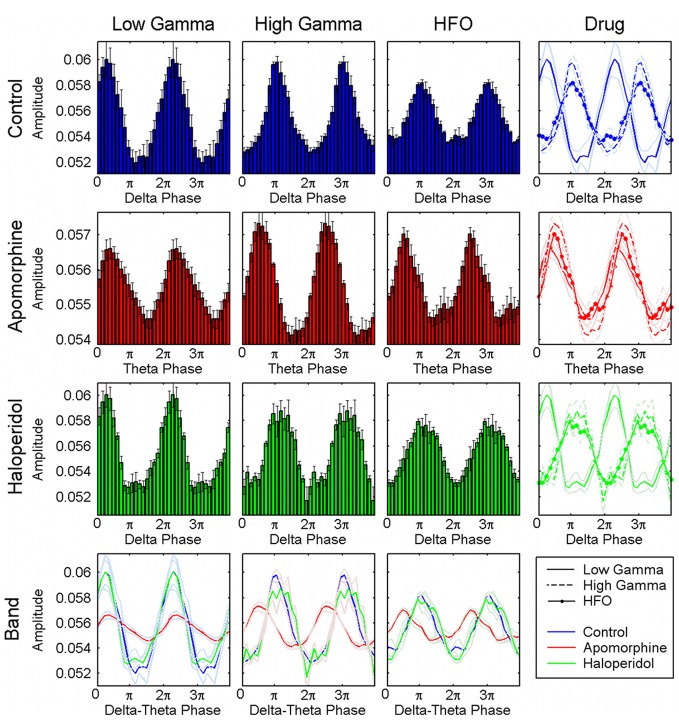
**Distribution of the preferred phases of coupling for the whole set of animals and structures.** Pooled results confirm the consistency of the preferred phase values across the different animals and structures analyzed. The first row shows in the first, second, and third panels the normalized amplitude of the low-gamma, high-gamma, and HFO oscillations related to the phase of the delta activity, respectively. The last panel displays the three the traces (together with their respective errors) superimposed and shows that the amplitude of HFO and high gamma oscillations is maximal at the troughs of the delta oscillations whereas low gamma peaks at the transition form the maxima to the descending phase of the delta cycle. The second row displays the same representation for the activities obtained under the effect of a apomorphine. Administration of this dopaminergic agonist shifts the preferred phase of coupling for the high-gamma and HFO simultaneously, and aligns them with that of the low-gamma oscillations (second row, last panel). Haloperidol does not induce any relevant changes in this coupling as shown in the third row. The last row displays the superimposed distributions of the normalized amplitudes for each of the three fast oscillations.

***Effects on the phase of coupling.*** Dopaminergic modulation also affected the preferred phase of the coupling patterns. in the control condition, high-gamma and hfo oscillations peaked at the troughs of the delta waves (Figures 4a, 5) with a pattern similar to that reported in previous studies of humans and rats (Canolty et al., 2006; Tort et al., 2008). by contrast, low-gamma oscillations peaked at the transition from maxima to the descending phases of the delta waves. apomorphine administration altered these patterns by modifying the value of the preferred theta-phase where high-gamma and hfo show their largest amplitudes (Figures 4b, 5). for all structures, the amplitudes of both gamma bands and hfo oscillations were maximal around the peak of the theta wave. this means that dopaminergic stimulation forces a simultaneous displacement of the preferred theta-phase for the high-gamma and hfo. haloperidol did not provoke any phase-shift changes with respect to the control condition (Figures 4c, 5). moreover, these patterns were very consistent across the whole set of animals and nuclei.

#### Temporal consistency of the PA-CFC pattern

**Figure 6:**
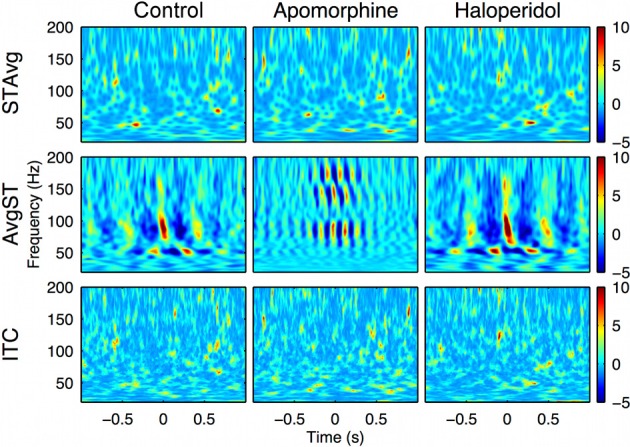
**Intra-structure evoked vs. induced changes in the oscillatory activity.** Delta-theta phase trough triggered changes in the oscillatory activity. First row: Stockwell transform of the averaged evoked potential (STAvg). No significant response was evoked (phase-locked) in the 20–200 Hz range relevant to the delta-theta phase. Second row: Average of the magnitude of single-sweep ST (AvgST). The delta-theta phase induced (non phase-locked) significant changes in low-gamma, high-gamma, and HFO bands in the three conditions. Third row: No significant Inter-trial coherence (ITC, a pure phase measure) values were detected using delta-theta phase troughs as a trigger. The results are obtained from the motor cortex recording of a single representative animal (rat 2).

To gain more insight about the precise timing of the interactions mediated by the PA-CFC mechanism, we computed the TF plot of the averaged raw signal obtained by aligning segments of the LFP traces to the troughs of the slow waves. Averaging single-sweep energy changes related to the troughs revealed the presence of consistent patterns of *induced (non-phase locked)* modulation of the gamma and HFO amplitudes, with a marked periodicity in the delta (theta) range (Figure 4, center row and Figure 6, second row). By contrast, no consistent *evoked (phase locked)* changes were detected when the phase of the gamma or HFO signals were taken into account (Figure 6, first row). This means that the phase of the slow frequency is parsing the timing of subsequent high frequency bursts, with a slight jitter among subsequent delta cycles. This effect was even more marked when computing a *pure phase measure*, the ITC, which only takes into account the phase of the signals (Figure 6, third row).

### Phase-amplitude CFC across the cortico-basal ganglia network

**Figure 7:**
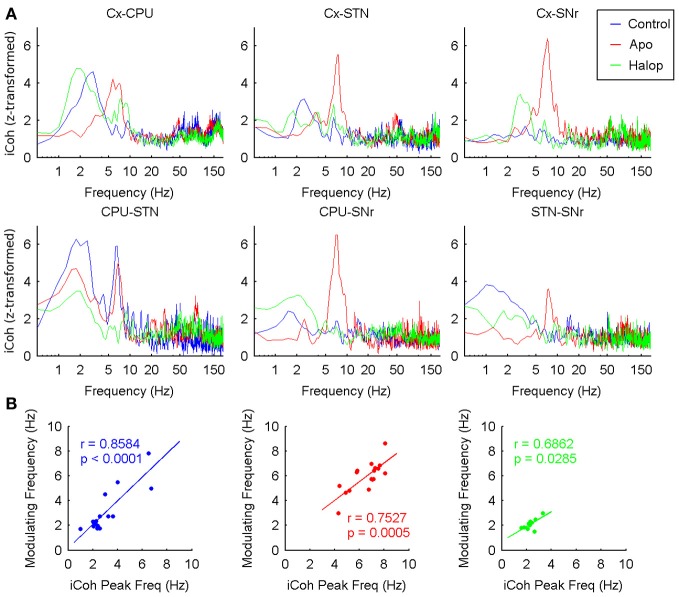
**Inter-structure interactions measured by means of imaginary coherence. (A)** Grand-Average of the z-transformed imaginary coherence values in the six possible pairs (Cx-CPU, Cx-STN, Cx-SNr, CPU-STN, CPU-SNr, and STN-SNr). Under saline and haloperidol conditions the strongest values of coherence are found in the delta band (~2 Hz), while after apomorphine injection the coherence is maximal in the theta range (~8 Hz). **(B)** Delta (theta) peak frequencies measured in the imaginary coherence spectrum are significantly correlated with the modulating frequency values across the different animals in the three conditions recorded.

The consistency of the PA-CFC patterns observed over the set of structures suggested the presence of complex interactions between nuclei. The values of iCoh for the six possible pairs of structures revealed the presence of significant relationships, mainly in the delta band following saline and haloperidol administration, and in the theta band after the administration of apomorphine (Figure 7A). Again, the frequency values of these peaks were correlated with those of the modulating activities detected in the PA-CFC co-modulograms (Figure 7B).

**Figure 8:**
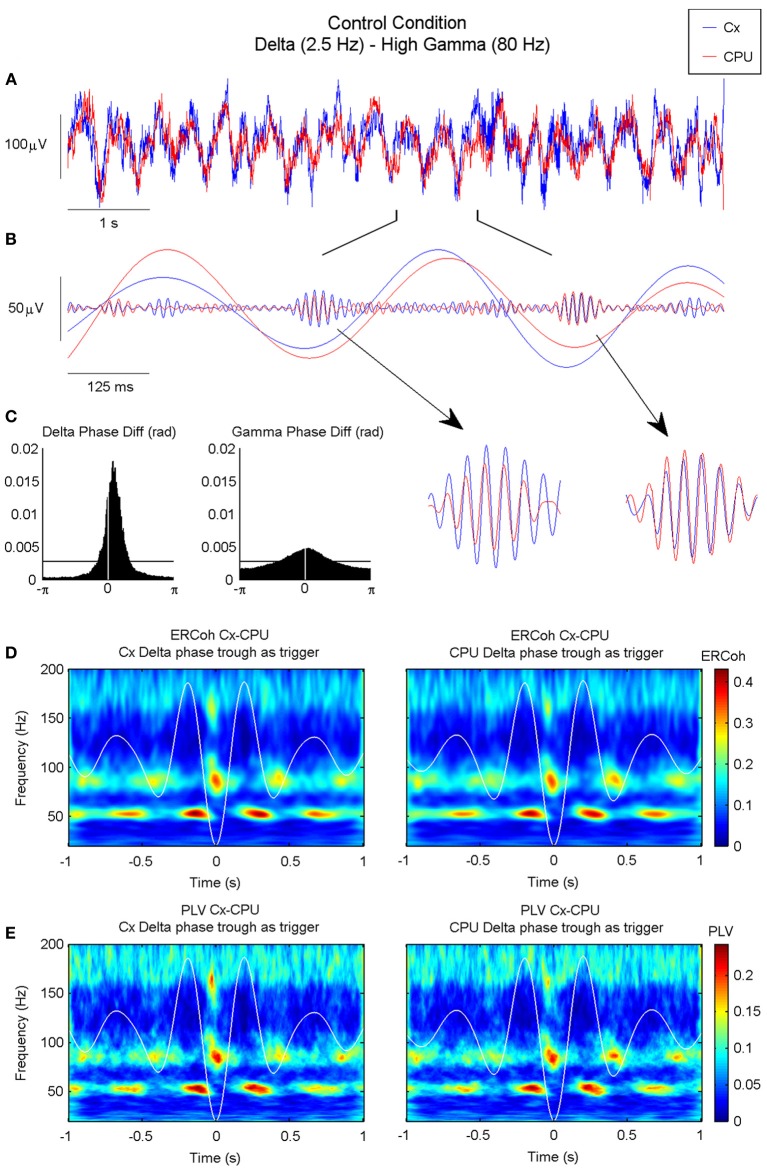
**Inter-structure gamma and HFO synchronization is mediated by delta oscillations in the motor circuit of the BG. (A)** Raw ECoG and LFP recorded in the motor cortex and CPU of a representative animal (rat 9), respectively. **(B)** Bandpass filtered oscillations of a 1 s fragment of **(A)** in which slow oscillations have been filtered in the delta range and fast oscillations in the high gamma range. Note that in the valley of the delta waves, both structures are highly synchronized in the high-gamma range (see detail). **(C)** Phase difference histograms show a non-zero phase difference in the delta band between the motor cortex and CPU, and a near-zero lag in the high-gamma band (the horizontal line indicates the mean level in an ideally random distribution). **(D–E)** Time-frequency maps representing the ERCoh **(D)** and PLV **(E)** between the motor cortex and CPU using the delta phase trough time points from both structures as triggers for the analysis (see methods).

Figure 8A shows an example of raw data signals recorded from the motor cortex and CPU simultaneously. Oscillations at different frequency ranges coexisted with a rich combination of delta oscillations, with activities at higher frequencies superimposed. Band-pass filtered versions of the signals showed that the two delta waves evolved simultaneously, while synchronous bursts of gamma activity rose at precise phases of these slow waves (Figure 8B). The histograms of phase differences revealed the existence of significant relationships across nuclei in both frequency ranges (see Figure 8C). In accordance with the iCoh analysis, both delta and gamma histograms showed unimodal distributions peaking at delays close, but not equal, to zero pointing out to the existence of lagged interactions within these two frequency bands. Together, these findings point toward the coexistence of cross-frequency and cross-nuclei mechanisms that could influence the organization of brain activity over different frequencies and temporal scales, both locally and across different structures. To explore this hypothesis, we used a bivariate analysis based on the event-related coherence scheme. By taking the delta troughs from one of the signals as the trigger for an ERCoh analysis we confirmed that the strength of the coherence within the low-gamma, high-gamma, and HFO bands was modulated by the phase of the delta waves. Again, a temporo-frequential organization was observed, whereby the coherence between low-gamma activities was maximal at the peak and descending phase of the delta waves, and the coherence in the high-gamma and HFO bands increased in the troughs of delta waves (Figure 8D). The use of a pure phase measure (PLV, see Methods) confirmed the robustness of this spatio-temporal organization (Figure 8E).

#### Effects of dopaminergic modulation

**Figure 9:**
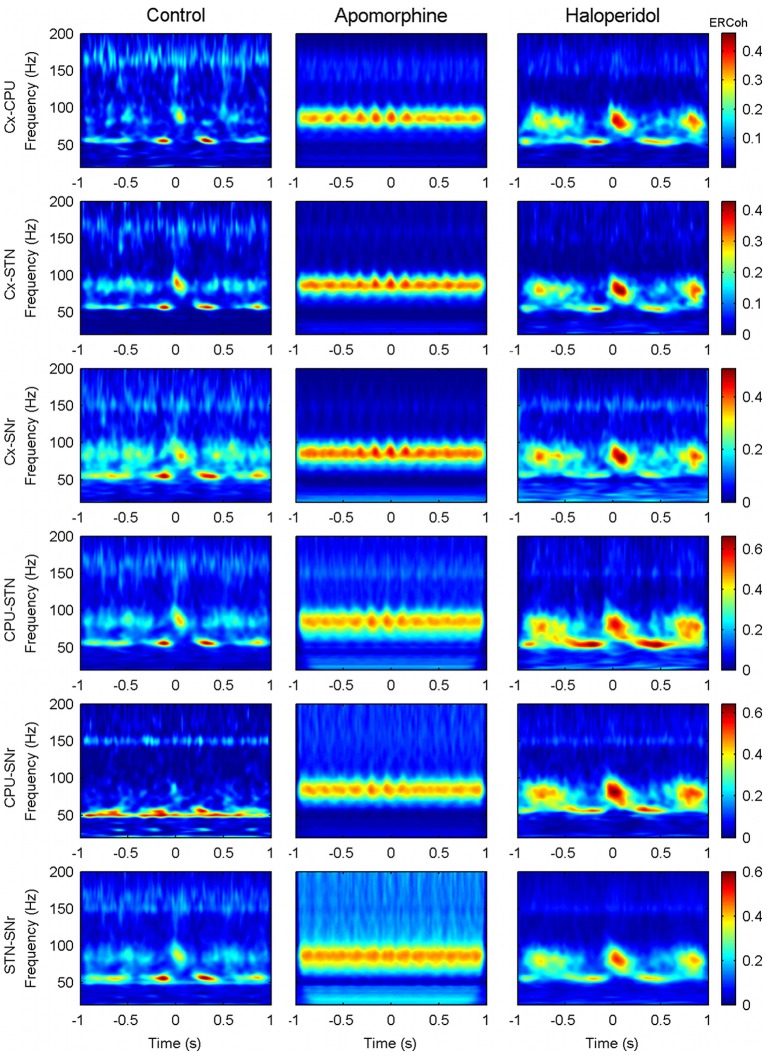
**Effect of dopaminergic modulation over the inter-structure patterns of CFC.** Panels show the delta (theta) triggered ERCoh maps in the six possible pairs (Cx-CPU, Cx-STN, Cx-SNr, CPU-STN, CPU-SNr, and STN-SNr) from a single representative animal (rat 5). For each pair of contacts the trigger is obtained by using delta-theta phase troughs from the first structure. Similar results were obtained when choosing the activity of the second structure to set the trigger (data not shown).

The same analysis was run for all possible pairs of structures and conditions. In the control condition and for all the different combinations of nuclei there was a strong modulation of the interactions in the low- and high-gamma bands and HFO, where periods of interaction alternated with periods with no interaction following delta-periodicity (see Figure 9, first column).

Under the effects of apomorphine (Figure 9, second column), the shift of the modulating frequency together with the increase in coupling of the theta/high-gamma pair determined a pattern of interaction that was mainly characterized by the existence of long lasting interactions in the high-gamma band that fluctuated at theta frequencies (effects in the low-gamma and HFO bands were also observed but to a lower extent).

The patterns after haloperidol administration (Figure 9, third column) were similar to those in the control condition in terms of the frequencies and periodicity of interactions. Nevertheless, an effect on the strength and duration of the interaction was observed. Accordingly, interactions persisted for longer compared to those observed in the control condition, mainly in the high-gamma band.

## Discussion

We report here three main results from multisite recordings in the cortico-basal ganglia network of freely behaving rats: (i) the amplitudes of low gamma (~50 Hz), high gamma (~80 Hz), and high frequency activities (~150 Hz) are coupled to the phase of delta (~3 Hz) waves; (ii) inter-site, transient synchronizations in the slow gamma, high gamma and HFO bands are determined by the phase of the delta waves; and (iii) manipulation of the dopaminergic system has a strong effect on this specific organization of brain rhythms.

### CFC in the cortico-basal ganglia network

Non-linear phase-amplitude coupling between different frequencies has been described as a physiological mechanism in sensory processing and memory circuits (Canolty et al., 2006; Lakatos et al., 2008; Tort et al., 2008; Cohen et al., 2009; Fujisawa and Buzsáki, 2011). In the hippocampus, a number of studies have shown that the phase of theta waves (8 Hz) modulates the amplitudes of activities in the low-gamma, high gamma, and HFO ranges (Bragin et al., 1995; Lakatos et al., 2005; Lisman, 2005; Canolty et al., 2006; Montgomery and Buzsáki, 2007; Tort et al., 2008). In the basal ganglia, CFC has been reported in both awake and anesthetized rodents (Berke et al., 2004; Mena-Segovia et al., 2008; Tort et al., 2008; Dzirasa et al., 2010; Jin and Costa, 2010; Dejean et al., 2011; Fujisawa and Buzsáki, 2011). Striatal activities in the theta band (3–8 Hz) dynamically modulate a narrow band of high-frequency oscillations in the 80–120 Hz range (Tort et al., 2008). We found a similar structure in the oscillations of the motor cortex, CPU, STN, and substantia nigra, with the phase of delta oscillations modulating activities in the low-gamma, high-gamma, and HFO frequency ranges. We found that high-gamma and HFO oscillations peaked at the troughs of the delta waves in the motor cortex and basal ganglia, as previously described in the striatum (Tort et al., 2008). However, we also found that low-gamma oscillations were maximal in the descending branch near the peak. This distinction between high and low-gamma oscillations is consistent with recent results reported in mice where neocortical recordings show selective theta phase modulation of neocortical rhythms during REM and distinguish high-gamma from low-gamma oscillations (Scheffzük et al., 2011). In the hippocampus, phase–amplitude interactions depend on both the frequency band and the CA1 layer in which the recordings are made (Tort et al., 2008; Colgin et al., 2009; Scheffer-Teixeira et al., 2012). All these findings suggest that in the neocortex, low-gamma (30–60 Hz), and high-gamma (60–90 Hz) waves are different and they result from independent physiological mechanisms with different functions. Indeed, high-gamma activities seem to be more related to HFO than to slow-gamma activities.

Amplitudes of high-gamma and HFO activities (but not low-gamma) were maximal near to the trough of the delta oscillation, both in the control condition and after haloperidol administration. Under the effect of apomorphine, we observed a shift on the preferred phase of these two frequency bands toward the theta peaks. Both gamma and HFO can be recorded in the human basal ganglia. Gamma oscillations in the basal ganglia have classically been associated with the “on” motor state (after dopaminergic medication) in PD patients (Alonso-Frech et al., 2006; Trottenberg et al., 2006). However, these oscillations seem to be widespread in the motor circuit, and a physiological role for them has been proposed (Jenkinson et al., 2013). HFO (~300 Hz) activity has also been recorded in the STN of parkinsonian and non-parkinsonian patients (Foffani et al., 2003; Danish et al., 2007; Lopez-Azcarate et al., 2010) and an exaggerated pattern of PA-CFC between the HFOs and the abnormal beta activity typical of the disease has been described in patients with PD (Lopez-Azcarate et al., 2010; Özkurt et al., 2011). Recently, an exaggerated pattern of coupling between the phase of this abnormal beta oscillation and the amplitude of higher frequencies (50–200 Hz) has also been detected in the primary motor cortex (M1) of PD patients (de Hemptinne et al., 2013) suggesting that also the motor cortex is restricted to a rigid pattern of coupling, avoiding it to react against signals coming from other brain regions.

### CFC across the cortico-basal ganglia network

Imaginary coherence analysis indicated that the largest degree of across-site interaction in the cortico-basal ganglia network was within the delta band (theta under apomorphine effects). Same-frequency, cross-structure interactions play a key role in the coordination of inter-site information (Crone et al., 2001; Edwards et al., 2005; Ray et al., 2008; Tort et al., 2008) and coherent low-frequency oscillations seem to be important in regulating large-scale networks (Benchenane et al., 2010; Canolty et al., 2010). We found that the frequencies with the largest inter-site interaction strength in the cortico-basal ganglia network are also the frequencies that mediate the PA-CFC patterns, thereby modulating the amplitudes of activities at higher frequencies. Time-lagged short-time bursts of coherent oscillations between structures in the gamma and HFO ranges were only detected in precise phases of the delta waves, suggesting that information is only transmitted at specific points in each cycle of the slow modulating activity, as hypothesized previously (Jensen and Colgin, 2007; Canolty and Knight, 2010). This precision in the timing of across-site oscillations contrasts with the absence of long-range temporal ITC observed at the local (intra-site) level.

While inter-structure, same-frequency delta-modulated interactions in the gamma and HFO lasted for seconds; intra-site delta-locked coherence maps vanished after a few milliseconds (i.e., some high frequency cycles), allowing for a fast reconfiguration of the rhythm’s structure. Dynamic modulation of CFC patterns has already been described in the rat striatum and hippocampus as transient PA-CFC patterns where the strength of the coupling changed over time and differed between structures (Tort et al., 2008). It was shown that CFC could span different areas, whereby the phase of the low frequency from the striatum affected the amplitudes of a specific range of high frequency oscillations in the hippocampus, and the phase of the hippocampal slow waves modulated the amplitude of other ranges of high frequency oscillations in the striatum. Similarly, it was shown that gamma oscillations in the hippocampus split into two different frequency bands that interact with different structures (Colgin et al., 2009). All these observations imply that CFC could serve also as a coupling mechanism, where low frequencies mediate simultaneous and possibly bidirectional communication using independent channels (Fries, 2009; Canolty and Knight, 2010). Here we have seen that the phase of delta activity could be responsible for gating the periods of transient communication between structures, thereby enabling or preventing the transfer of information. These patterns of inter/intra frequency coupling between multiple brain areas would have an impact on the spiking of single cells and they might help to bring together anatomically dispersed functional cell assemblies (Canolty et al., 2010). Alternatively, given that the communication windows open simultaneously, they might recruit coherently oscillating neuronal groups (Fries, 2005, 2009). As a result, intra-site delta-mediated CFC, together with inter-site delta coherence, may provide a type of spatio-temporal selection that could facilitate frequency-domain modularity and spatial selectivity for the exchange of information across distant structures, while maintaining a sort of non-linear, local hierarchy in terms of frequency. Indeed, recently it has been shown that in PD patients the amplitude of cortical (M1 area) gamma activity is modulated by de anomalous beta activity recorded from the STN, suggesting an effect of cortical oscillations in maintaining the pathological basal ganglia oscillations (de Hemptinne et al., 2013).

### Effect of dopaminergic stimulation

We found that the modulation of the dopaminergic system exerts a substantial effect over the PA-CFC patterns and therefore, in the organization of the oscillatory activity across the cortico-basal ganglia network. The main effect was a significant frequency shift of the whole PA-CFC pattern observed in the control conditions. Apomorphine increased the animals’ locomotion and shifted the modulating frequency from the delta to the theta range. On the contrary haloperidol lowered these frequency values (although it did not exert significant effects on the total locomotion). Thus, drugs with inverse effects over the dopaminergic system, caused opposite variations in the frequency of the modulating waves. Although the observed changes in LFP oscillations and their interactions could be partially affected by changes in locomotion induced by the dopaminergic drugs, correlations were only significant under the effect of apomorphine and for very specific frequencies.

A role of the dopaminergic system in the generation of theta hippocampal oscillations has been proposed (Fujisawa and Buzsáki, 2011). Moreover, transient inactivation of the VTA decreases hippocampal theta power (Yoder and Pang, 2005) and VTA stimulation increases theta burst firing of medial septal neurons, mediated by D1/5 receptors (Fitch et al., 2006). A pacemaker role of the VTA is compatible with these observations and might be even a source of theta oscillations in meso-limbic system (Fujisawa and Buzsáki, 2011). In PD patients, repetitive movements and temporal perception are abnormal but can be normalized by dopaminergic drugs, suggesting that dopaminergic neurons might act as a “pacemaker” and facilitate rhythm generation (Artieda et al., 1992). Different aberrant oscillatory patterns have been reported in PD (Brown, 2003; Alonso-Frech et al., 2006) and in the case of the exaggerated PA-CFC coupling between the abnormal beta activity and the HFOs (~300 Hz), dopaminergic treatment induces changes in the CFC together with an improvement in the motor state (Lopez-Azcarate et al., 2010; Özkurt et al., 2011). In addition, it has been shown how deep brain stimulation of the STN in PD patients suppresses the exaggerated cortical PA-CFC patterns in the primary motor area (de Hemptinne et al., 2013). These findings suggest that non-linear coupling between frequencies may not only play a physiological role (as shown previously), but that it might also participate in the pathophysiology of Parkinsonism or other brain disorders.

### Final remarks

A fundamental fact to assess the functional relevance of CFC is whether it facilitates the modulation or the timing of spike discharges. The most detailed studies on this topic have been carried out in the rat hippocampus (Lisman and Jensen, 2013). It has been proposed that the phenomenon called *phase precession* could be dependent on theta-gamma coupling (O'Keefe and Recce, 1993). According to this hypothesis, assemblies of place cells (pyramidal neurons in the rat hippocampus that discharge in relationship with the spatial location of the animal) discharge in a single gamma cycle within a preferred phase range of each theta cycle. As there is “room” for several gamma cycles within each theta cycle, the gamma cycle associated to the discharge of each particular assembly can occupy different “slots,” earlier or later depending on the position of the animal. The precision in this temporo-spatial coding would occur thanks to the theta-gamma PA-CFC, that facilitate the discharge timing of the place cells creating temporal windows with higher probability of action potential discharge (Csicsvari et al., 1999; Buzsáki and Moser, 2013).

In the cortico-basal ganglia network, recent reports suggest that the anomalous CFC patterns detected in M1 and STN of PD could be related to the exaggerated synchronization of the action potentials of basal ganglia neurons (de Hemptinne et al., 2013; Shimamoto et al., 2013). Simultaneously recorded basal ganglia neurons show pathological firing patterns synchronized in the beta range (Mallet et al., 2008). As neurons also tend to be synchronized to the LFPs recorded in the same structure, basal ganglia neurons are likely to promote spiking patterns with similar rhythmicities on the structures where they project. In LFP recordings, these unitary responses could be detected as a contributor of the high frequency oscillations and thus, resulting in the detected CFC patterns (Buzsáki et al., 2012). Nevertheless, and although the ultimate mechanisms that give origin to the oscillatory activity and the CFC are not known, in general terms it is thought that the damaged dopaminergic projections to the striatum and cortex might be causing a change in the spiking dynamics of the neurons and their interrelationships.

The results presented here provide new insights into the functional organization of the oscillatory activity in the cortico-basal ganglia network. We found that low-frequency entrainment combined with phase-amplitude CFC may provide a mechanism to co-ordinate faster activities (and thus neuronal responses) across different frequency bands and spatial structures. This specific spatio-temporal organization is modified by dopaminergic drugs, which indicates a physiological role for the dopaminergic system in this mechanism and suggests a potential pathophysiological explanation for the effects of dopaminergic deficits as those observed in pathologies like PD.

### Conflict of interest statement

The authors declare that the research was conducted in the absence of any commercial or financial relationships that could be construed as a potential conflict of interest.
